# Immunodominant T Cell Determinants of Aquaporin-4, the Autoantigen Associated with Neuromyelitis Optica

**DOI:** 10.1371/journal.pone.0015050

**Published:** 2010-11-30

**Authors:** Patricia A. Nelson, Mojgan Khodadoust, Thomas Prodhomme, Collin Spencer, Juan Carlos Patarroyo, Michel Varrin-Doyer, Joseph D. Ho, Robert M. Stroud, Scott S. Zamvil

**Affiliations:** 1 Department of Neurology, University of California San Francisco, San Francisco, California, United States of America; 2 Department of Biochemistry, University of California San Francisco, San Francisco, California, United States of America; Julius-Maximilians-Universität Würzburg, Germany

## Abstract

Autoantibodies that target the water channel aquaporin-4 (AQP4) in neuromyelitis optica (NMO) are IgG1, a T cell-dependent Ig subclass. However, a role for AQP4-specific T cells in this CNS inflammatory disease is not known. To evaluate their potential role in CNS autoimmunity, we have identified and characterized T cells that respond to AQP4 in C57BL/6 and SJL/J mice, two strains that are commonly studied in models of CNS inflammatory diseases. Mice were immunized with either overlapping peptides or intact hAQP4 protein encompassing the entire 323 amino acid sequence. T cell determinants identified from examination of the AQP4 peptide (p) library were located within AQP4 p21-40, p91-110, p101-120, p166-180, p231-250 and p261-280 in C57BL/6 mice, and within p11-30, p21-40, p101-120, p126-140 and p261-280 in SJL/J mice. AQP4-specific T cells were CD4^+^ and MHC II-restricted. In recall responses to immunization with intact AQP4, T cells responded primarily to p21-40, indicating this region contains the immunodominant T cell epitope(s) for both strains. AQP4 p21-40-primed T cells secreted both IFN-γ and IL-17. The core immunodominant AQP4 21-40 T cell determinant was mapped to residues 24-35 in C57BL/6 mice and 23-35 in SJL/J mice. Our identification of the AQP4 T cell determinants and characterization of its immunodominant determinant should permit investigators to evaluate the role of AQP4-specific T cells in vivo and to develop AQP4-targeted murine NMO models.

## Introduction

Neuromyelitis optica (NMO) is a rare, aggressive, often fatal, inflammatory demyelinating disease that predominantly affects the optic nerves and spinal cord [1], [2], [3]. For many years, NMO was considered a subtype of multiple sclerosis (MS). However, it is now recognized that a majority of NMO patients develop autoantibodies (NMO-IgG) against aquaporin 4 (AQP4) [4]. AQP4 is the predominant water channel in the central nervous system and is localized in astrocyte foot processes, but can also be found in other organs, including kidneys, heart and lungs. Therefore, restricted inflammation of NMO cannot be solely explained by the pattern of expression of AQP4. AQP4-specific antibodies in NMO patients are IgG1 [5], an antibody subclass that requires help from antigen (Ag)-specific CD4^+^ T cells [6], [7]. Although little is known regarding recognition of AQP4 by T cells, AQP4-specific T cells may drive the humoral AQP4-specific immune response.

While AQP4 is the most characterized candidate autoantigen in NMO, it is not known whether an immune response that selectively targets AQP4 initiates the pathologic changes associated with NMO. Development of animal models for NMO should permit direct assessment of immune responses in vivo. A "Devic-like" disease, characterized by spontaneous optic neuritis and paralysis associated with spinal cord inflammation occurred when myelin oligodendrocyte glycoprotein (MOG)-specific T cell receptor (TCR) transgenic (Tg) mice were crossed to MOG-specific B cell receptor (BCR) knock-in mice [8], [9]. However, the autoantigen was MOG, and not AQP4. Models that target AQP4 have met with modest success [10], [11], [12]. For example, it was observed that AQP4-specific antibodies promoted NMO-like pathologic changes within the CNS of rats with experimental autoimmune encephalomyelitis (EAE), a myelin-specific T-cell mediated CNS inflammatory disease. However, these pathologic changes did not occur in the absence of T-cell mediated inflammation, suggesting that T cells may have an important role in NMO pathogenesis.

Our goal in this investigation was to characterize T cell recognition of AQP4. In this regard, we have examined AQP4 T cell reactivity in C57BL/6 (H-2^b^) and SJL/J (H-2^s^) mice, two strains that are susceptible to various models of autoimmune neurologic disease [13], [14], [15], including the CNS demyelinating disease EAE [13]. We evaluated T cell reactivity by immunizing mice with individual overlapping 15 and 20 amino peptides from a library encompassing the entire AQP4 sequence, and tested recall to these peptides. To characterize the naturally processed AQP4 determinants, we have immunized mice with intact AQP4 and tested recall to AQP4 peptides. Here, we report on the identification of several discrete AQP4 T cell determinants and the characterization of the naturally processed immunodominant determinants.

## Results and Discussion

### Identification of AQP4 T cell determinants

**Figure 1:**
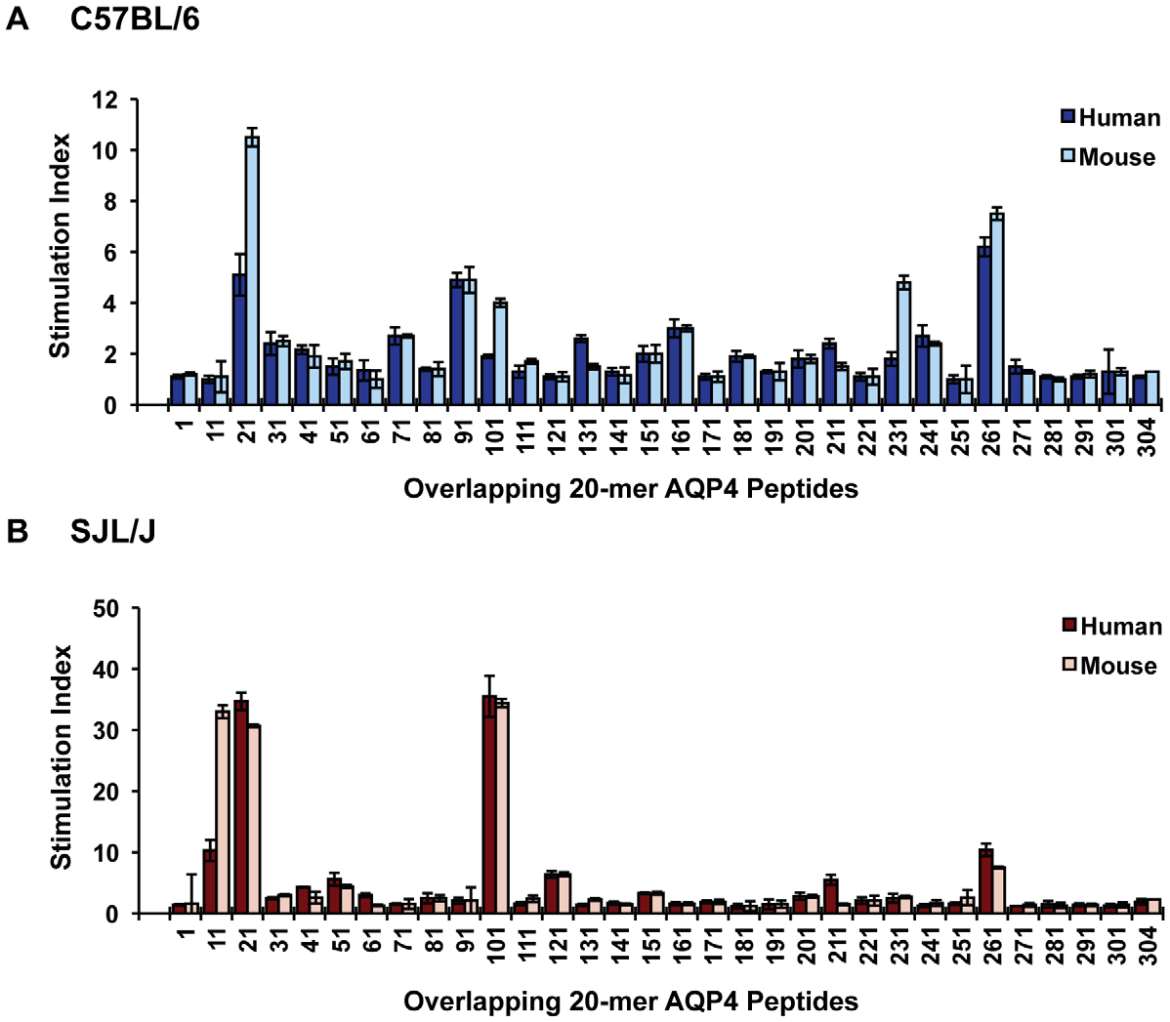
Identification of AQP4 peptides that elicit T cell responses. AQP4 peptides were tested for their capability to induce T cell proliferative responses in (A) C57BL/6 and (B) SJL/J mice. Each overlapping 20-mer AQP4 peptide is indicated by first residue. Mice were immunized subcutaneously with 100 µg murine AQP4 20-mer peptides in CFA. 10–12 days later, lymph node cells were isolated and tested in vitro for recall responses to the mouse or corresponding human peptides used for immunization. Data are shown as stimulation indices (SI's) of mean proliferative responses in the presence of peptide (25 µg/ml) compared to the absence of antigen (background). Standard errors (+/− SEM) are shown for proliferative responses tested in triplicate.

**Figure 2:**
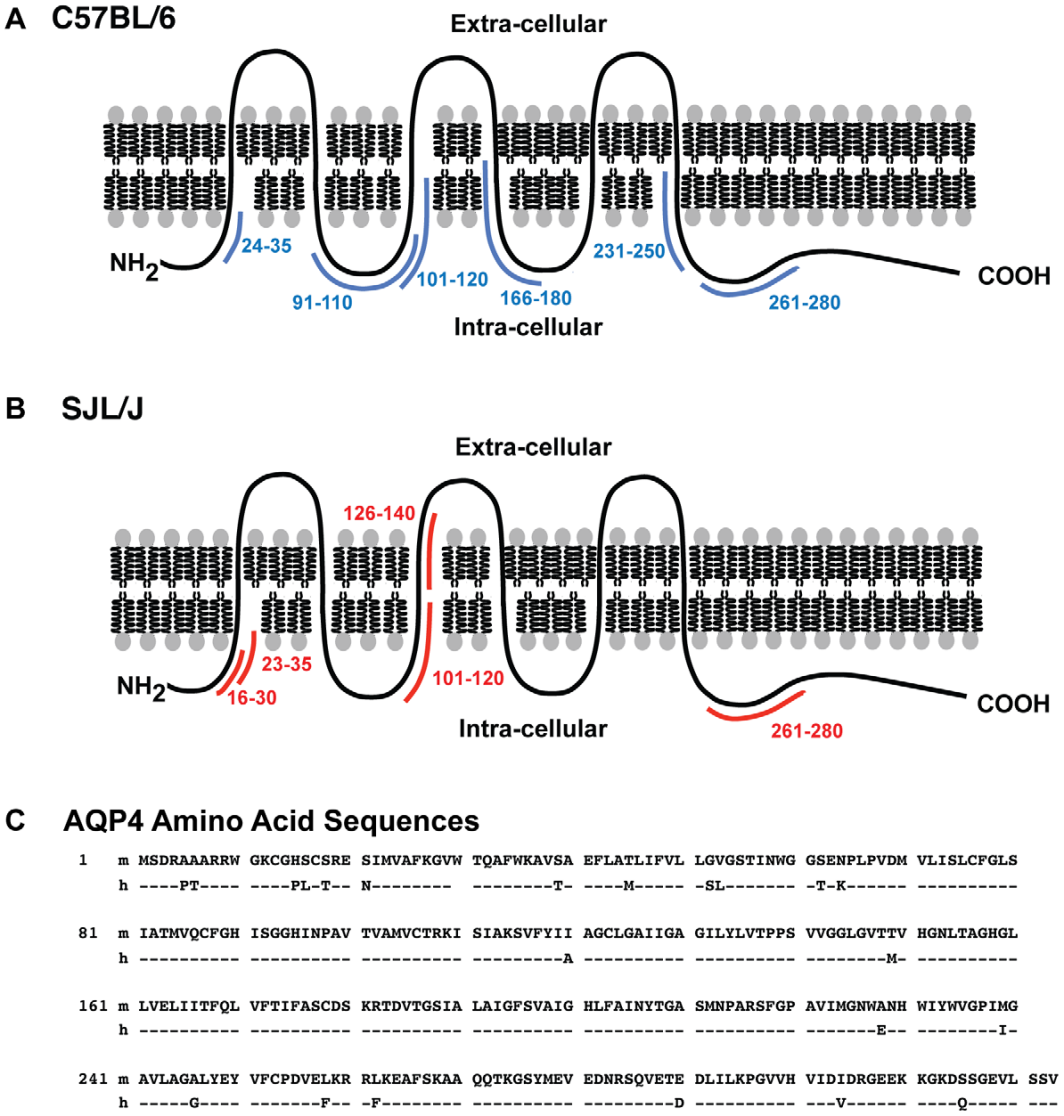
Localization of identified murine AQP4 T cell epitopes. AQP4 peptides that elicited proliferative responses in (A) C57BL/6 and (B) SJL/J mice are located within putative transmembrane and cytoplasmic domains. (C) Sequences of human (hAQP4) [34] and murine [17] AQP4 (mAQP4). Dashes represent homologous regions between the two species.

In contrast to B cells and their secreted antibodies, which frequently recognize conformational determinants, Ag-specific CD4^+^ T cells recognize specific Ag determinants in association with MHC II molecules expressed on the cell surface of Ag presenting cells (APCs) [16]. We first identified the immunogenic regions of AQP4 by testing proliferative T cell responses to individual peptides from a library of overlapping 15-mers and 20-mers encompassing the entire AQP4 sequence. By direct immunization of C57BL/6 mice with murine AQP4 peptides, we identified five regions of AQP4 that contain T cell determinants: p21-40, p91-110, p101-120, p161-180, p231-250 and p261-280 (Figure 1A). Of these peptides, proliferative responses to p21-40, p91-110, p161-180 and p261-280 were more robust. AQP4 T cell determinants in SJL/J were located within p11-30, p21-40, p101-120, 126-140 and p261-280 (Figure 1B). T cells that proliferated to AQP4 were CD4^+^ and MHC II-restricted (data not shown). All AQP4 T cell determinants in C57BL/6 and SJL/J mice were found within putative transmembrane or cytoplasmic domains **(** Figure 2). Specifically, we did not identify T cell determinants within the three extracellular domains, the A, C, and E loops [17], which may contain B cell determinants recognized by AQP4-specific antibodies from NMO patients [18].

### AQP4 p21-40 is the naturally processed immunodominant determinant for recognition by CD4^+^ T cells

**Figure 3:**
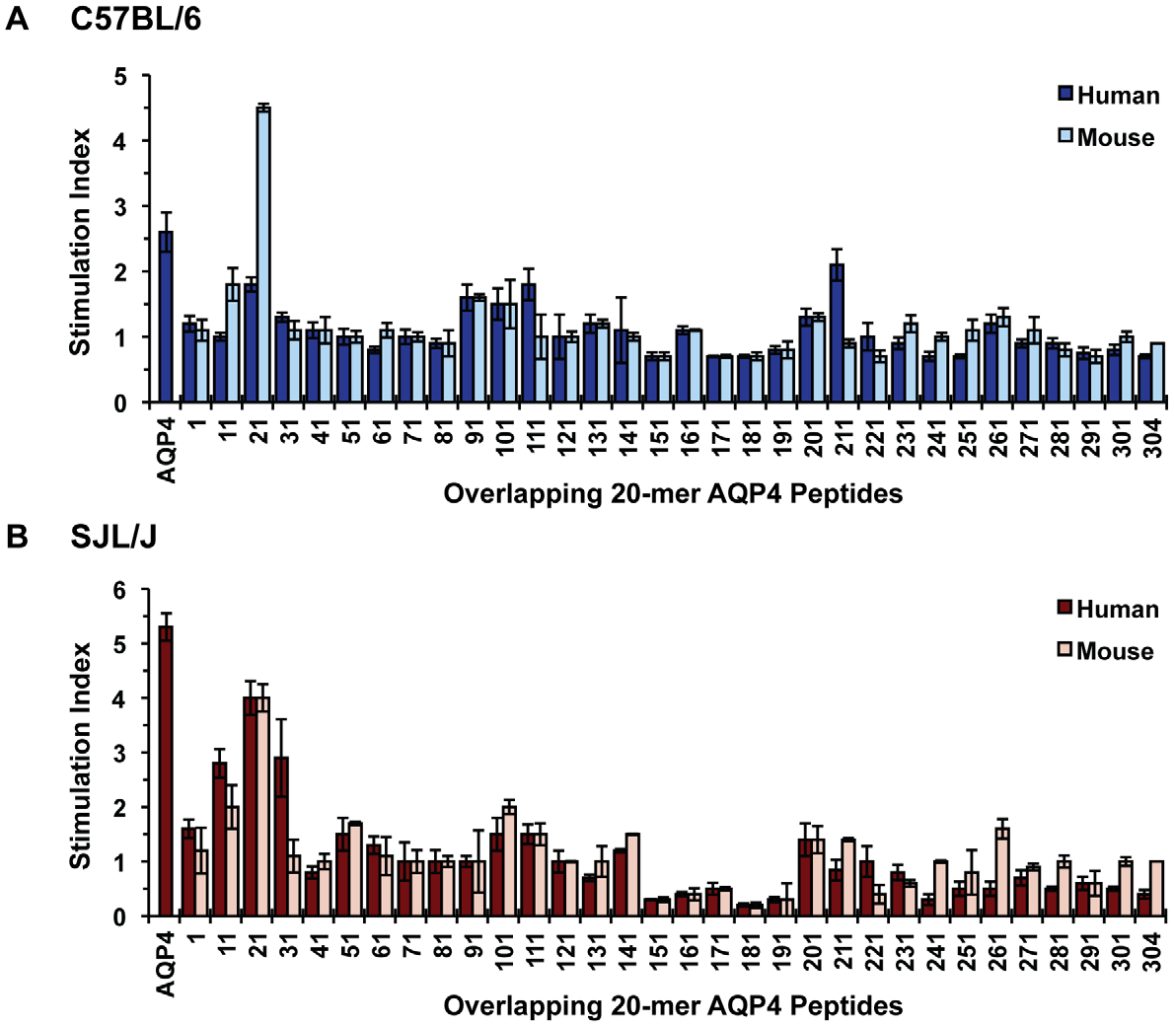
Identification of naturally processed AQP4 determinants. AQP4 peptides were tested for their ability to induce T cell proliferative responses in intact hAQP4-primed (A) C57BL/6 and (B) SJL/J mice. Each 20-mer AQP4 peptide is indicated by first residue. Mice were immunized subcutaneously with 100 µg recombinant intact hAQP4 in CFA. 10–12 days later, lymph node cells were cultured in vitro for recall responses to the indicated human or mouse overlapping 20-mer peptides. Data are shown as stimulation indices (SI's) of mean proliferative responses in the presence of peptide (25 µg/ml) compared to the absence of antigen (background). Standard errors (+/− SEM) are shown for proliferative responses tested in triplicate. Recall to intact hAQP4 is shown for comparison.

**Figure 4:**
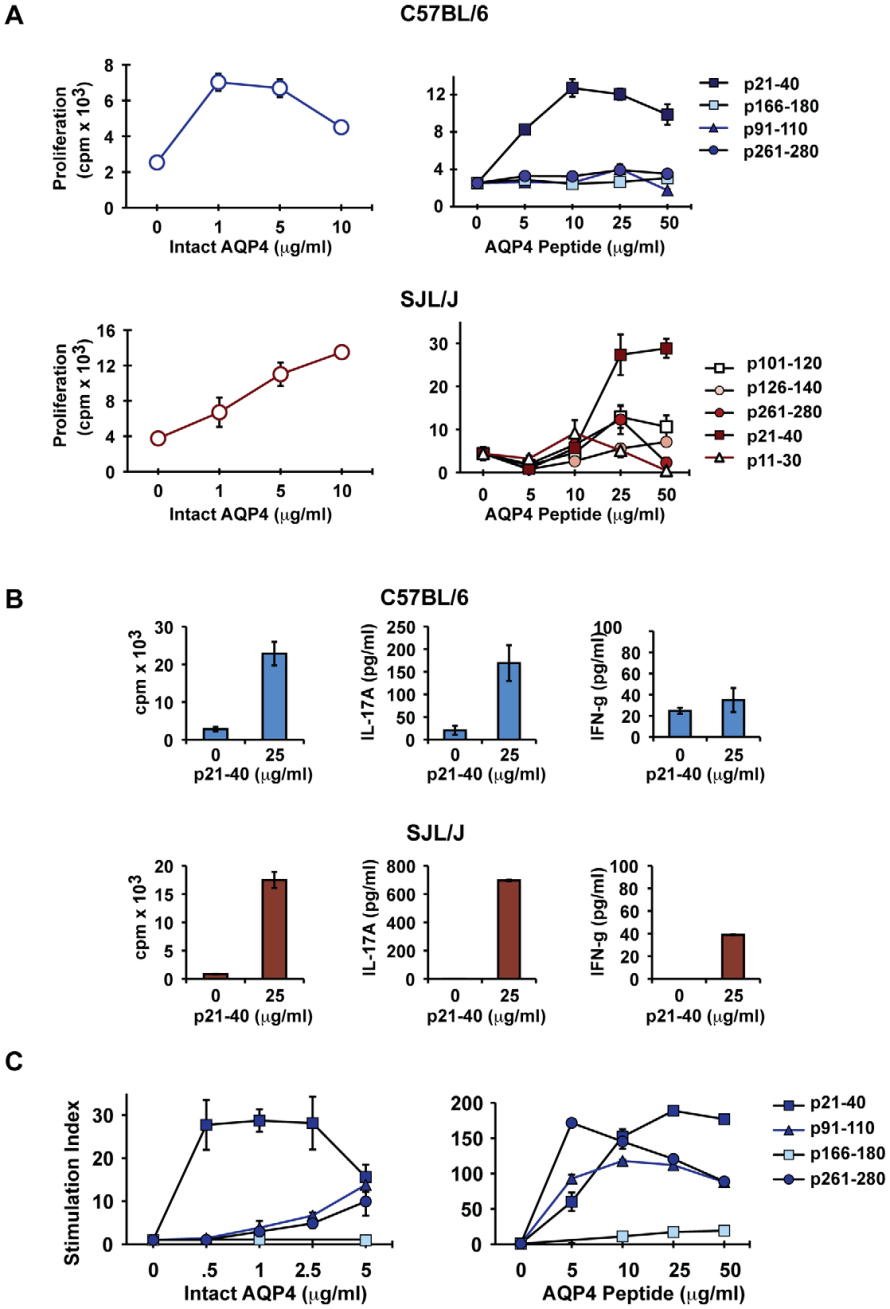
AQP4 p21-40 is a naturally processed immunodominant determinant of intact AQP4. (A) C57BL/6 (above) and SJL/J (below) mice were immunized subcutaneously with 100 µg recombinant intact hAQP4 in CFA. Lymph nodes were harvested at day 10–12 and cultured in the presence of various concentrations of either intact hAQP4 (left), or individual murine AQP4 peptides (right) for 4 days. Proliferation was measured by ^3^H-thymidine incorporation, and are presented as mean cpm +/− SEM for triplicates. (B) C57BL/6 (above) and SJL/J (below) m21-40 primed lymph node cells were assayed for cytokine production and proliferation in response to m21-40 peptide. Supernatants were collected after 72 hr for IFN-γ and IL-17A for ELISA. Data are presented as mean +/− SEM for triplicates. (C) Proliferation of C57BL/6 T cell lines specific to p21-40, p91-110, p166-180, and p261-280 were assayed following re-stimulation with various concentrations of intact hAQP4 (left) and self-peptides (right). Data are shown as stimulation indices (SI's) of triplicates of antigen proliferation over no antigen conditions (background).

Although peptide fragments can bind cell surface MHC molecules directly and elicit T cell immune responses, presentation of native Ag by APCs to CD4^+^ T cells in vivo usually requires processing, which involves proteolytic degradation of protein within APCs and association of peptide cleavage products with MHC II molecules [19], [20]. Thus, in parallel with our analysis by direct immunization of AQP4 peptides, C57BL/6 and SJL/J mice were separately immunized with intact AQP4, and tested for recall to intact AQP4 and the individual overlapping AQP4 peptides. Using this approach, we could identify the naturally processed immunodominant AQP4 determinants. Intact human AQP4, which is 93% homologous to mouse AQP4 [17] (Figure 2), was used as the immunogen. Immunization with recombinant human AQP4 elicited significant proliferation to itself in both strains (Figure 3), although the magnitude was lower in comparison to the stimulation induced by the most immunogenic AQP4 peptides (Figure 1). For the negative controls, no response was found to either MOG 35-55 (C57BL/6) or PLP 139-151 (SJL/J) after AQP4 immunization (data not shown). In both strains, a significant recall response was detected to mouse AQP4 p21-40 (Figure 3 and 4A), indicating that this is a naturally processed determinant of intact AQP4. T cells that recognized AQP4 p21-40 secreted IFN-γ and IL-17, indicating that both Th1 and Th17 immune responses were elicited (Figure 4B). Our findings also suggest that peptides p91-110, p101-120, p166-180, p231-250 and p261-280, which elicited responses in C57BL/6 mice (Figure 1), and p11-30, p101-120, p126-140 and p261-280, which elicited responses in SJL/J mice, were not efficiently processed (Figure 3). To further examine this possibility, we generated T cell lines to these AQP4 peptides. In contrast to AQP4 p21-40-specific T cells, other AQP4 peptide-specific T cell lines proliferated in response to their respective AQP4 determinants, but less efficiently, or not at all, to intact AQP4 (Figure 4C). Collectively, our results indicate that AQP4 p21-40 contains a naturally processed immunodominant AQP4 determinant.

### T cell epitope specificity within the immunodominant AQP4 p21-40

**Figure 5:**
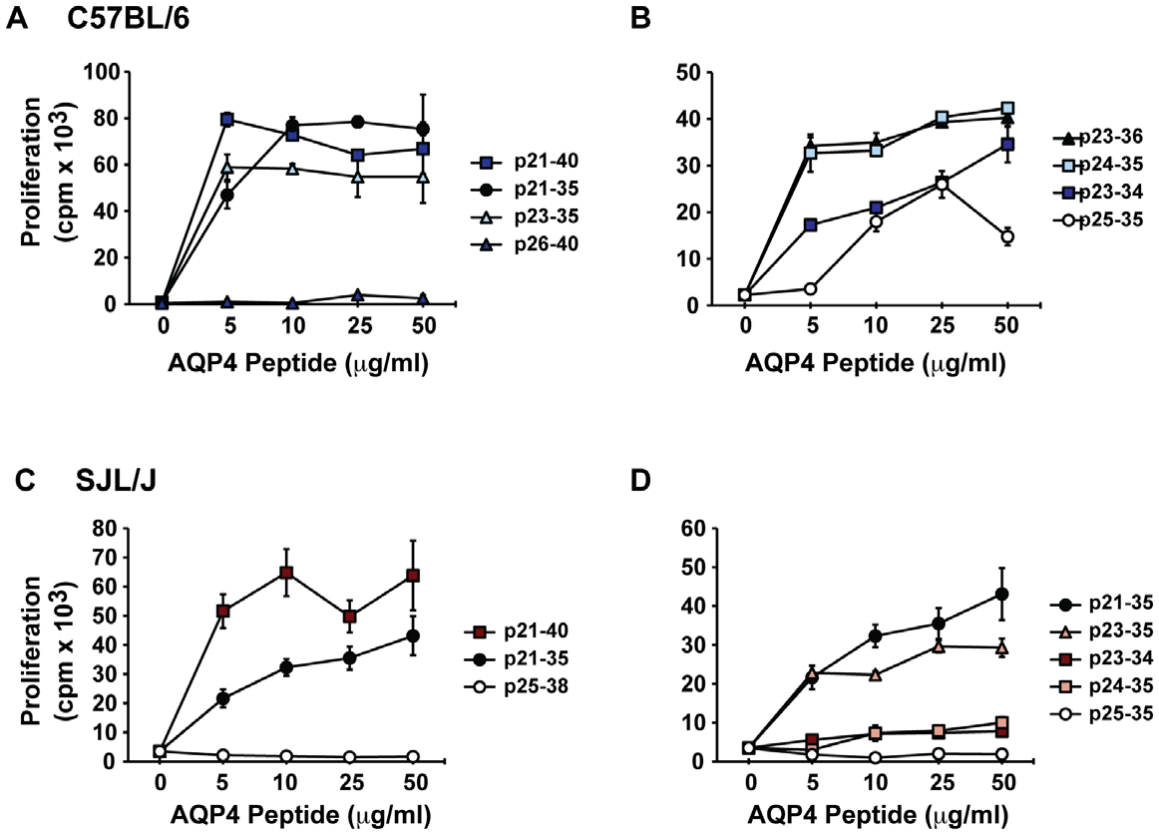
Characterization of the minimal core T cell epitope within AQP4 p21-40. AQP4 p21-40-specific T cells were restimulated with truncated peptides to determine the core of the p21-40 determinant in (A, B) p21-40 specific C57BL/6 cell lines and (C, D) p21-40 specific SJL/J primary lymph node cells. Cell lines were restimulated with irradiated syngeneic splenic APC and various concentrations of p21-40 or truncated peptides. After 48 hours (cell lines) or 72 hours (LN), cultures were pulsed with ^3^H-thymidine and harvested 16 hours later. Data shown represent means of triplicates +/− SEM.

In general, MHC II-restricted CD4^+^ T cells recognize 9-13 amino acid peptides [16], [20]. In order to further characterize the immunodominant AQP4 T cell epitope(s), synthetic truncated peptides corresponding to sequences within AQP4 21-40 were synthesized and tested for recognition by p21-40-specific T cells. AQP4 p21-40-specific T cells from C57BL/6 mice proliferated in response to p21-35, p23-35 and p24-35, but not to p26-40 (Figure 5A and B). Shorter AQP4 peptides, p25-35 and p23-34 also stimulated proliferation of p21-40-specific T cells, although less efficiently. These results indicate that p24-35 is the core immunodominant AQP4 T cell determinant in C57BL/6 mice.

AQP4 p21-40-specific T cells from SJL/J mice also recognized p21-35, but neither p25-38 (Figure 5C and D), nor p26-40 (data not shown). Those T cells responded nearly as efficiently to AQP4 p23-35 as to p21-35, but did not respond to p24-35 or p25-35, demonstrating that the immunodominant AQP4 T cell determinant in SJL/J mice is p23-35. Thus, our data suggest that the immunodominant T cell epitopes for C57BL/6 and SJL/J mice are located in the same 13 amino acid sequence, p23-35, although the fine specificities are not identical.

It is notable that the amino acid residues with the immunodominant determinant are important in AQP4 functions [21], [22]. Residues 24-26 are critical for the assembly of orthogonal arrays of particles (OAPs), which are thought to enhance water transport [21]. AQP4 residues 23-32 also contain the site required for the interaction with the regulator glutamate receptor 5 (mGluR5) and the catalytic subunit of Na,K-ATPase in forming macromolecular microdomains that may participate in K^+^ homeostasis [22]. Although intriguing, T cell recognition of this same region of AQP4 is probably coincidental, since determinants recognized by CD4^+^ T cells are generally created through processing and association with MHC II molecules [16]. Further, unlike professional APCs, including microglia and dendritic cells, astrocytes, which express AQP4, are not efficient APCs in vivo [23].

While mouse and human AQP4 are 93% homologous, it is recognized that small differences in amino acid sequences, e.g. single residue substitutions, in heterologous proteins can have important effects on MHC binding as well as TCR-mediated stimulation [24], [25]. However, three of the immunogenic AQP4 peptides that we identified, p91-110 and p166-180 in C57BL/6 mice (Figure 1A), and p126-140 in SJL/J mice (Figure 1B), share identical sequences between mouse and human AQP4 (Figure 2C). Further, we observed cross-reactivity for most immunogenic AQP4 peptides that were not entirely homologous. Mouse AQP4 p21-40, which has xenogenic substitutions at residues 21 and 39, stimulated T cell responses to both itself and human AQP4 p21-40, and mouse p21-40-specific T cell lines were also stimulated by human intact AQP4 (Figure 4C). Our findings clearly indicate that AQP4 21-40 is an immunodominant region of AQP4 in both strains.

**Figure 6:**
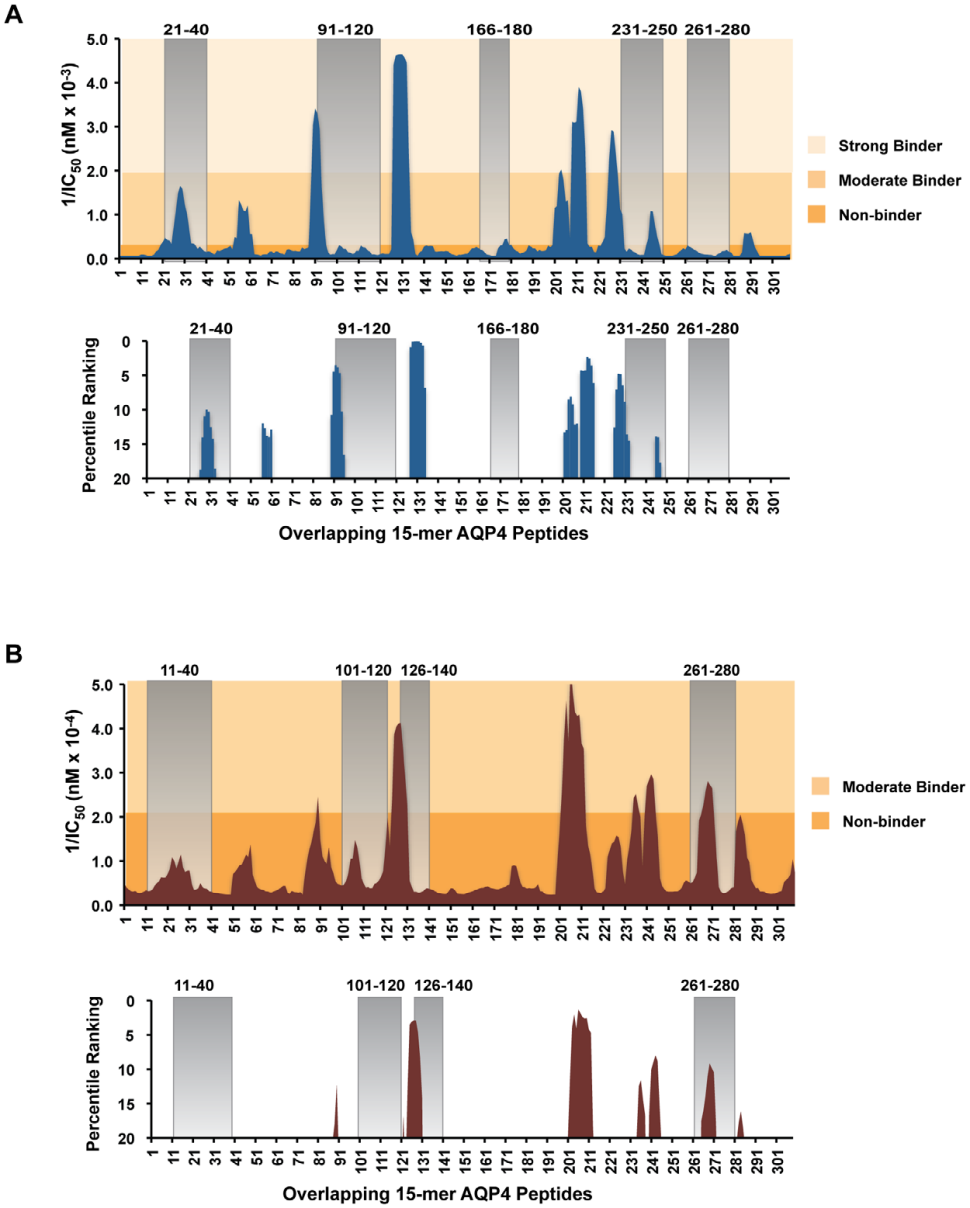
Predicted I-A^b^- and I-A^s^- binding mAQP4 epitopes for T cell recognition. MetaSVMp, a quantitative binding affinity program that employs the immune epitope database (IEDB) [26], [27], [30], was used to identify potential I-A^b^- and I-A^s^-binding of AQP4 determinants for T cell recognition. Inverse of 50% inhibition concentration values (1/IC_50_) is plotted for I-A^b^ (A top panel) and I-A^s^ (B top panel). Peaks correspond to increased predicted binding affinity: strong binding, IC_50_<500 nM; moderate binding, IC_50_>500 nM and <5,000 nM, and non-binding, IC_50_>5,000 nM. Peaks correspond to increased predicted binding affinity. The top 20% of all possible epitopes are shown for I-A^b^ (A bottom panel) and I-A^s^ (B bottom panel). MetaSVMp percentile ranks were acquired from the MetaMHC web-based application; tall peaks correspond to top-ranked predicted epitopes.

In this investigation, we have identified immunogenic T cell determinants using a library of overlapping peptides encompassing the entire sequence of AQP4. While this approach may be considered labor-intensive, several different *in silico* methods have recently been developed in order to efficiently identify potential MHC-restricted T cell determinants [26], [27], [28], [29]. Many of these programs base predictions upon existing quantitative peptide binding affinities for specific MHC molecules [26], [27], [28], [29]. In general, methods for predicting MHC I-restricted determinants have advanced more rapidly, in part due to the fact that MHC II has an open binding groove accommodating larger peptides. For most programs available, there is better poor resolution for I-A^b^ (Figure 6A) than for I-A^s^ (Figure 6B), possibly reflecting the larger database of known I-A^b^-restricted T cell determinants of Ag. MetaSVMp, a program that utilizes relative affinities of identified determinants from the immune epitope database (IEDB) to predict MHC II binding peptides [26], [27], [30], identified eight distinct regions of AQP4 that contain potential determinants that bind I-A^b^ for T cell recognition (Figure 6A top panel). AQP4 p21-40 ranked among the top 12 percentile of predicted I-A^b^-binding AQP4 15-mers (Figure 6A bottom panel). However, MetaSVMp did not predict T cell recognition of AQP4 166-180 or 261-280. MetaSVMp also predicted the AQP4 sequence 121-140 within the top one percentile, yet we did not detect proliferation to this peptide either by recall to direct peptide immunization or in response to intact AQP4 in C57BL/6 mice. Therefore, our results caution against relying solely upon using predictive algorithms for identification of T cell epitopes of proteins.

The presence of T cells within active NMO lesions [2], evidence for clonal T cell expansion in peripheral blood of NMO patients [31], and the observations that AQP4-specific antibodies from NMO patients were not pathogenic in rats in the absence of CNS inflammation provide indirect evidence suggesting that T cells may participate in the etiology of NMO. Knowing that AQP4-specific antibodies in NMO serum are T-cell dependent IgG (IgG1) points to antigen-specific CD4^+^ T cell-mediation of its humoral response. However, there are several questions regarding the location and nature of AQP4-specific T cells in NMO pathogenesis. Do AQP4-specific T cells direct the AQP4-specific immune response outside the CNS? Do AQP4-specific T cells participate directly in CNS inflammation? Serum NMO IgG concentrations are higher than CSF levels [32], indicating that development of AQP4-specific antibodies occurs outside the CNS and is driven by stimulation within the peripheral immune compartment. Observations that encephalitogenic MBP-specific T cells promoted the development of NMO-like lesions upon transfer of AQP4-specific antibodies provided evidence that inflammation initiated by a T cell immune response to a CNS myelin antigen was sufficient for entry of AQP4-specific antibodies, and questions whether the cellular response that initiates CNS inflammation in NMO must necessarily target AQP4.

Elevated levels of IFN-γ and IL-17 have also been detected within the CSF of NMO patients [33], supporting the existence of both Th1 and Th17 responses. Active NMO lesions are characterized by the abundance of eosinophils and neutrophils [2], which suggests that Th2 and Th17 cells may have a key role in the CNS inflammation. While it may not be feasible in clinical studies to assess whether AQP4-specific T cells participate directly in NMO pathogenesis, now that we have discovered the T cell determinants of AQP4, it should permit investigators to determine the relative contribution of these subsets of AQP4-specific T cells in the initiation of CNS inflammatory responses in models. Efforts in developing a murine AQP4-based NMO model should focus on the AQP4 T cell epitopes identified, and in particular to the N-terminal region. The identification of regions of AQP4 recognized in mice may also provide insights regarding recognition of human AQP4 by NMO patients.

## Materials and Methods

### Mice

C57BL/6 (H-2^b^) and SJL/J (H-2^s^) female mice, 8 weeks of age, were purchased from the Jackson Laboratories (Bar Harbor, ME) and housed under specific pathogen-free conditions at UCSF Laboratory Animal Research Center. **Ethics Statement:** The experimental protocol adheres to guidelines for animal use in research set by the National Institutes of Health and was approved by the Office of Research, UCSF Institutional Animal Care and Use Committee (IACUC Approval AN083156-01).

### Antigens

AQP4 15-mer and 20-mer peptides were synthesized by Genemed Synthesis Inc. (San Antonio, TX) according to the sequences for mouse [17] and human AQP4 proteins [34]. Overlapping 20-mer peptides were offset by ten amino acids. Peptides corresponding to certain hydrophobic AQP4 sequences were synthesized in overlapping 15-mer peptide pairs: 41-55 & 46-60, 121-135 & 126-140, 151-165 & 156-170, 161-175 & 166-180, 191-205 & 196-210, and 221-235 & 226-240. All peptides were greater than 95% pure by HPLC analysis, and were reconstituted using 100% sterile DMSO (Sigma-Aldrich, St. Louis, MO) to a concentration of 20 mg/ml for stock solution. Full length recombinant (r) human AQP4 (1-323) was expressed in *Pichia pastoris* and purified as described [35]. Mouse myelin oligodendrocyte glycoprotein (MOG) peptide 35-55 and proteolipid protein (PLP) 139-151 were synthesized by Auspep (Parkville, Australia). Following identification of relevant epitopes, independent synthesis of candidate peptides was undertaken by Auspep (Parkville, Australia).

### Proliferation Assays

Mice were immunized subcutaneously with 100 µg intact hrAQP4 or murine AQP4 peptides in CFA. Lymph nodes were harvested at day 10–12 and 2×10^5^ cells/well were cultured in 96 well plates in the presence of various concentrations of AQP4, the same murine peptide, or the corresponding human peptide, for 4 days. Responses to PLP or MOG were used as negative controls. Data are presented as counts per minute (cpm) ^3^H-thymidine incorporated for triplicate wells. Stimulation Index (SI) was calculated by dividing cpm in wells with Ag by cpm in wells with no antigen controls of each assay test group.

### T cell lines

Mice were immunized subcutaneously with 100 µg hrAQP4 or murine AQP4 peptides in CFA. Lymph nodes were harvested at day 10–12, strained through a sterile 70 µM mesh filter. Cells were washed and plated at a concentration of 5×10^6^ cells per well in 24 well plates with 25 µg/ml Ag in complete RPMI medium. Lines were subsequently pushed with antigen at 25 µg/ml and irradiated syngeneic spleen cells every 14 days, and assayed for proliferative response to Ag and AQP4 in triplicate.

### Cytokine analysis

Supernatants for cytokine analysis were collected at 72 h after stimulation in culture with Ag as previously described for lymph node cells. Undiluted supernatants from triplicate culture wells were assayed for cytokines using ELISA Ready Set Go kits from eBioscience for both IFN-γ and IL-17A using NUNC Maxisorp 96 well ELISA plates. ELISA readouts were visualized with SoftMax Pro 4.3.1 LS software and Spectramax Plus 384 (Molecular Devices Corporation, Sunnyvale, CA) and plotted for triplicates +/− SEM.

### Statistical analysis

Data are presented as mean ± standard error of mean (SEM) for triplicates. Stimulation Index (SI) was calculated using the ratio of means of triplicate experimental conditions to background (no antigen) controls. Standard errors for experiments using SI refer to deviations among triplicate conditions over mean background proliferation. All other statistical analysis was performed using a one-way multiple-range analysis of variance test (ANOVA) for multiple comparisons. Two-way ANOVA was performed for analysis of peptide recall for T-cell line assays taking both peptide used and concentration of peptide as parameters for analysis.

### MHC II epitope prediction by quantitative analytical programs

Quantitative binding affinity servers were used to predict MHC II-restricted epitopes of mAQP4 in C57BL/6 (H-2^b^) and SJL/J (H-2^s^) mice. Web-based applications used for prediction for murine alleles included MHCPRED (http://www.darrenflower.info/mhcpred/) [28], [36], NetMHCII (I-A^b^ only) (http://www.cbs.dtu.dk/services/NetMHCII/) [29], IEDB (http://tools.immuneepitope.org/) [27] and MetaMHC (http://www.biokdd.fudan.edu.cn/Service/MetaMHC.html) [26]. MetaSVMp, an algorithm from the MetaMHC application, is a stacked generalization that combines the base predictors for SMM-align [30] and local alignment (LA) kernel [37], [38] into a support vector machine. The entire murine AQP4 (M1 isoform) sequence was entered for analysis of overlapping 15 amino acid peptides when applicable. Results are given as values of IC_50_ (50% inhibition concentration) from programs available for quantitative binding prediction, as well as percentile rankings within algorithms. The inverse of these IC_50_ values (1/IC_50_) is plotted in parallel with the top 20% of predicted binding peptides for MetaSVMp.
